# GLS-1, a Novel P Granule Component, Modulates a Network of Conserved RNA Regulators to Influence Germ Cell Fate Decisions

**DOI:** 10.1371/journal.pgen.1000494

**Published:** 2009-05-22

**Authors:** Agata Rybarska, Martin Harterink, Britta Jedamzik, Adam P. Kupinski, Mark Schmid, Christian R. Eckmann

**Affiliations:** Max Planck Institute of Molecular Cell Biology and Genetics (MPI-CBG), Dresden, Germany; University of Chicago, United States of America

## Abstract

Post-transcriptional regulatory mechanisms are widely used to influence cell fate decisions in germ cells, early embryos, and neurons. Many conserved cytoplasmic RNA regulatory proteins associate with each other and assemble on target mRNAs, forming ribonucleoprotein (RNP) complexes, to control the mRNAs translational output. How these RNA regulatory networks are orchestrated during development to regulate cell fate decisions remains elusive. We addressed this problem by focusing on *Caenorhabditis elegans* germline development, an exemplar of post-transcriptional control mechanisms. Here, we report the discovery of GLS-1, a new factor required for many aspects of germline development, including the oocyte cell fate in hermaphrodites and germline survival. We find that GLS-1 is a cytoplasmic protein that localizes in germ cells dynamically to germplasm (P) granules. Furthermore, its functions depend on its ability to form a protein complex with the RNA-binding Bicaudal-C ortholog GLD-3, a translational activator and P granule component important for similar germ cell fate decisions. Based on genetic epistasis experiments and in vitro competition experiments, we suggest that GLS-1 releases FBF/Pumilio from GLD-3 repression. This facilitates the sperm-to-oocyte switch, as liberated FBF represses the translation of mRNAs encoding spermatogenesis-promoting factors. Our proposed molecular mechanism is based on the GLS-1 protein acting as a molecular mimic of FBF/Pumilio. Furthermore, we suggest that a maternal GLS-1/GLD-3 complex in early embryos promotes the expression of mRNAs encoding germline survival factors. Our work identifies GLS-1 as a fundamental regulator of germline development. GLS-1 directs germ cell fate decisions by modulating the availability and activity of a single translational network component, GLD-3. Hence, the elucidation of the mechanisms underlying GLS-1 functions provides a new example of how conserved machinery can be developmentally manipulated to influence cell fate decisions and tissue development.

## Author Summary

Germ cells differ from somatic cells in their unique potential to reproduce a multicellular organism. The immortal germ line links the successive generations in all metazoans, but its development is remarkably diverse. How germline development and survival are regulated in different organisms is far from understood. One fundamental similarity is the widespread use of post-transcriptional mRNA regulation to control the expression of germ cell fate determinants. The development of the *C. elegans* germ line is a paradigm in the study of translational regulatory networks, composed of conserved RNA-binding or modifying proteins that act as mRNA regulators. Here, we report the discovery of GLS-1, a novel cytoplasmic protein, which we find to form a protein complex with the translational activator GLD-3/Bicaudal-C. This complex promotes and maintains the sperm-to-oocyte switch in hermaphrodites, whereby GLS-1 acts as a molecular mimic of FBF/Pumilio, a translational repressor of sperm promoting mRNAs. Furthermore, a GLS-1/GLD-3 complex may also positively regulate mRNAs important for germline survival. Therefore, GLS-1 serves as a new example of how cell fate decisions and tissue development are achieved by modulating the activities of broadly operating translational control networks.

## Introduction

Germ line and early embryonic gene expression rely largely on cytoplasmic mRNA control mechanisms, allowing for maximum flexibility of control [1]. A striking example is the unique ability of germ cells to transiently differentiate into gametes before forming a totipotent zygote upon fertilization. Many conserved cytoplasmic RNA-binding and RNA-modifying proteins have been found to support germline development, by associating with mRNA molecules in RNP complexes. In higher eukaryotes, these *trans*-acting factors can form larger RNP aggregates, termed germplasm granules [2]–[4]. Although these RNPs are anticipated to confer germ cell identity and are important for germline development their developmental regulation is largely unknown. Furthermore, it remains to be determined how these RNP complexes are utilized in an organism-specific fashion to control protein synthesis, i.e. the mRNA's translational output.

In nematodes, components of germplasm granules (P granules) are deposited maternally to the embryo and segregate to germ cell precursors, which suggests their early requirement for germline function. One such conserved maternal component is the Bicaudal-C (Bic-C) protein family member GLD-3 [5]. Bic-C proteins are involved in poly(A) tail metabolism of mRNAs [6],[7]. The *gld-3* locus encodes two major protein isoforms, GLD-3L and GLD-3S, of which both form a cytoplasmic poly(A) polymerase complex with GLD-2 [8]. Similar to *Drosophila* Bic-C, which is required for oogenesis and patterning of the embryo, GLD-3 is required for many aspects of germline development and embryogenesis, including a role in germline sex determination and germline survival [5],[9],[10].

The *C. elegans* sperm-to-oocyte switch serves as a paradigm for the analysis of post-transcriptional mRNA regulation [11]. A sex determination pathway determines the sperm and oocyte fate. Although hermaphrodites develop somatically as females, they produce a limited number of sperm during their fourth larval stage, before switching to continuous oocyte production in the adult. Therefore, the female sex determination pathway has to be temporarily suppressed to facilitate spermatogenesis. The underlying molecular mechanism is based on multiple interconnected RNA regulators, e.g. Bic-C, PUF, and Nanos proteins, that together comprise a molecular switch to regulate the timely accumulation of first sperm and then oocyte promoting factors. Interestingly, members of these RNA regulatory protein families are broadly conserved and seem to be utilized in other, yet less well understood, cell fate decisions [11].

Two counteracting forces balance the translational output of the key male fate promoting factor, *fem-3* [12]; GLD-3L acts as a translational activator whereas two very similar PUF (Pumilio and FBF) proteins, FBF-1 and FBF-2, collectively referred to as FBF, are translational repressors of *fem-3* mRNA. FBF-mediated repression of FEM-3 protein synthesis promotes oogenesis indirectly and is aided by a physical interaction with NOS-3, a worm Nanos ortholog [13],[14]. Yet to allow sperm production, in males and temporarily in the L4 hermaphrodite larvae, FBF's oogenesis-promoting activity has to be blocked. This is achieved by zygotic GLD-3L, which reduces FBF's affinity for its cognate regulatory element in the *fem-3* mRNA by binding to FBF's RNA-binding domain [5]. However, in order to switch to oogenesis, FBF must then be activated by a currently unknown mechanism.

These conserved RNA regulators are also involved in the less understood cell fate decision of germ cell survival [11]. In zygotes where GLD-3 is not supplied by the mother, germ cells are correctly specified during embryogenesis but degenerate during postembryonic development. Thus importantly, maternal *gld-3* activity is required to prevent germ cell degeneration [5]. Consistent with a role in germline development is that maternal GLD-3 is associated with P granules in the early embryonic P lineage (P1–P4). This also suggests a role for other P granule components in germline maintenance [5]. Additionally, little is known about the roles and interactions of various Nanos and PUF proteins that are required for germline maintenance in *C. elegans*, *Drosophila* and mice [13], [15]–[17]. Therefore, we reasoned that further insights into the controls of this cell fate decision might be more easily gained by focusing on the interactions of the single most important player conferring germ cell survival, GLD-3.

In this paper, we report the identification and characterization of *gls-1* (***g***erm***l***ine ***s***urvival defective-1). The *gls-1* gene activity is required for multiple aspects of germline development, including the sperm-to-oocyte switch and germline survival. The GLS-1 protein was identified in a yeast two-hybrid screen for GLD-3 interactors, and is a novel P granule component. We demonstrate that during germ cell fate decisions, GLS-1 exerts its roles by modulating GLD-3 activities. We provide evidence that GLS-1 interferes with GLD-3L inhibition of FBF/Pumilio, to assist the translational repression of spermatogenic factors. Furthermore, we find that the maternal GLS-1/GLD-3 complex prevents adult germ cell degeneration. We propose the GLS-1/GLD-3 complex accomplishes this by translationally activating mRNAs encoding survival factors required in the early embryonic germ cell lineage.

## Results

### GLS-1 Is a Novel Protein and Expressed in the Germ Line

**Figure 1:**
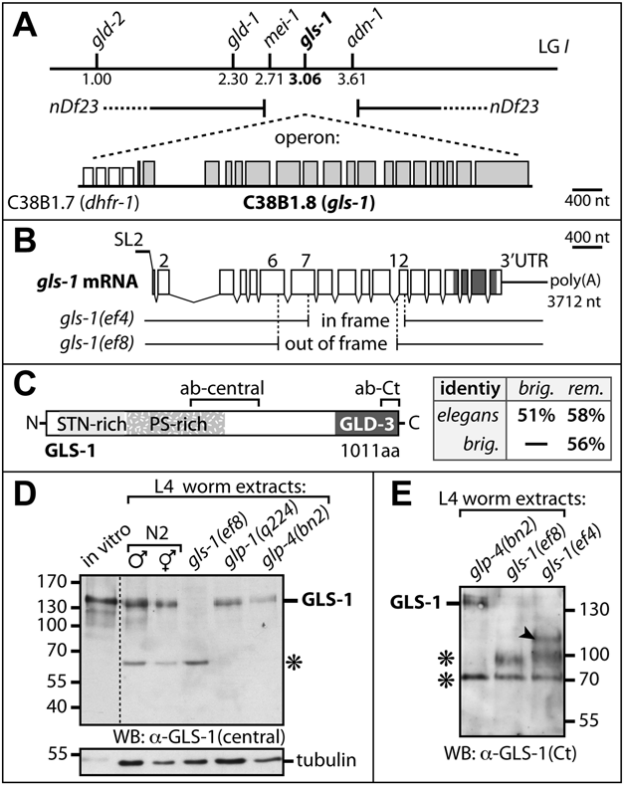
The *gls-1* Genomic Locus and Gene Products. (A) Genetic map position. *gls-1* is a downstream gene in an operon with *dhfr-1* on chromosome (LG) I. The deficiency *nDf23* removes genes in the approximate interval of +2 to +4 including *gls-1*. Exons, boxes. (B) *gls-1* transcripts and deletion mutants. *gls-1* mRNAs are SL2 spliced and vary slightly in their coding potential in the first exon by 6 nts (not shown). Coding exons are shown in boxes connected by thin lines representing introns. The encoded GLD-3 interaction domain is indicated. Extent of the 1657 bp *gls-1(ef8)* and 1253 bp *gls-1(ef4)* deletions is indicated by gaps. (C) GLS-1 protein structure. Fragments for antibody (ab) generation, the serine/threonine/asparagine-rich (STN), the proline/serine-rich (PS) and the minimal GLD-3 interaction region are outlined. GLS-1 conservation among three *Caenorhabditis* species is given in percent. *brig*, *briggsae*; *rem*, *remanei*. (D–E) Immunoblots of protein extracts prepared from indicated genotypes. *glp-1(q224ts)* and *glp-4(bn2ts)* animals with essentially no germ line raised at 25°C. His-tagged recombinant GLS-1 (in vitro) is given as a size reference. GLS-1 was detected in (D) with the central polyclonal antibody and in (E) with the C-terminal monoclonal antibody. A truncated GLS-1 form (arrowhead) of the expected size is present in *gls-1(ef4)* mutant animals. Asterisks mark non-specific background bands, which serve as loading controls (150 worms each lane).

Using the yeast two-hybrid system we aimed to identify new interaction partners of GLD-3. Several partial cDNAs of the gene C36B1.8, which we have named *gls-1*, were repeatedly found in two independent screens (Text S1). In our analysis of the *gls-1* genomic locus we found full-length (fl) *gls-1* cDNAs *trans*-spliced to SL2, suggesting a transcriptional co-regulation with the upstream gene C36B1.7 (Figure 1A,B; Text S1). We termed C36B1.7, *dhfr-1*, as its predicted ORF encodes a gene similar to dihydrofolate reductase.

The *gls-1* locus encodes a novel protein with no predictable functional motifs. The amino(N)-terminus of GLS-1 contains two consecutive regions, one rich in serines/threonines/asparagine and one rich in serines/prolines (Figure 1C). The GLD-3 interaction domain is located at the very carboxy(C)-terminus. Although no proteins similar to GLS-1 could be identified in vertebrates we identified the *gls-1* locus in *C. briggsae* and *C. remanei* where it is also in synteny with the upstream gene *dhfr-1* (Text S1). All GLS-1 proteins of the *Caenorhabditis* clade share approximately 55% amino acid sequence identity when compared to each other. The two shaded regions in the N-terminus in Figure 1C are conserved to almost 80% identity. We conclude that *gls-1* is a conserved nematode gene, co-transcribed with *dhfr-1*.

To study the GLS-1 protein, we raised several anti-GLS-1 sera. Two rabbit polyclonal antisera were generated using the central and C-terminal part of GLS-1, respectively (Figure 1C). Both rabbit antisera were affinity purified and recognized a recombinant His-epitope tagged GLS-1(FL) of ∼150 kDa (Figure 1D). A band of similar size was observed in protein extracts prepared from wild-type L4 animals of either sex but not from *gls-1(ef8)* deletion mutants, which delete the GLS-1 epitope (Figure 1D). Furthermore, GLS-1 expression is reduced in animals that essentially lack a germ line at 25°C, *glp-1(q224ts)* and *glp-4(bn2ts)* (Figure 1D). We conclude that GLS-1 is expressed in the germ line and soma.

### GLS-1 Is Cytoplasmic and Localizes to P Granules in Germ Cells

**Figure 2:**
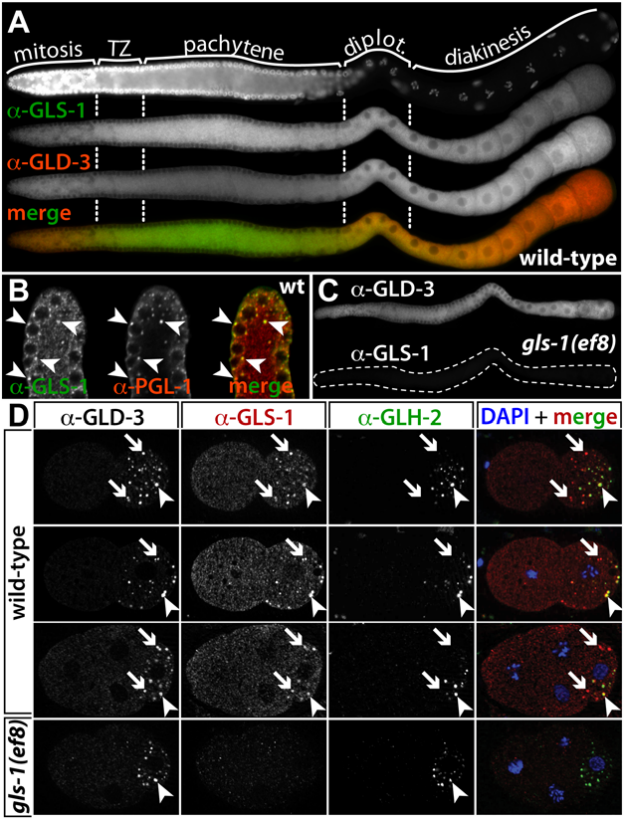
GLS-1 Expression in the Hermaphrodite Germ Line and Embryos. (A) GLS-1 profile in a wild-type adult germ line. Monoclonal anti-GLS-1 staining and epi-fluorescence microscopy. GLS-1 is expressed throughout the germ line and overlaps with GLD-3 expression. diplot., diplotene; TZ, transition zone. (B) Confocal images of a distal tip of a wild-type germ line. GLS-1 (green), detected with the polyclonal antibody. Its granular pattern overlaps (arrowheads) with P granules, marked by PGL-1 (red). Note, some P granules stain strongly for GLS-1 and weakly for PGL-1. (C) No immunoreactivity of the monoclonal GLS-1 antibody is seen in *gls-1(ef8)* mutant germ lines (outlined). The GLD-3 pattern appears unchanged in *gls-1(ef8)*. (D) In early wild-type embryos maternal GLS-1 localizes to P granules. Monoclonal anti-GLS-1 stainings. Optical sections of epi-fluorescence images taken with an Apotome (Zeiss). 2-cell embryo (two different focal planes given, top two rows) and 4-cell embryo (third row). 4-cell embryo of a *gls-1(ef8)* mother (bottom). Anterior, left; posterior, right. Note, granules strongly enriched for GLS-1 and GLD-3 and less so for GLH-2 (arrow), granules enriched for GLS-1, GLD-3 and GLH-2 (arrowhead).

To address GLS-1 expression in germ cells we performed immunocytochemistry on extruded germ lines. We either used the central rabbit antibody, pre-blocked with *gls-1(ef8)* protein extract, or a monoclonal anti-GLS-1 antibody, raised against the C-terminus of GLS-1. Germ cells and P granules were visualized with antibodies to the P granule component PGL-1 [18] or GLH-2 [19]. GLS-1 is expressed in all germ cells, with the exception of spermatocytes (not shown). In the adult hermaphrodite, GLS-1 is present in the mitotic region, accumulates during early stages of meiotic prophase I and is slightly less abundant in maturing proximal oocytes (Figure 2A). In addition to a diffused cytoplasmic localization, we observe a granular GLS-1 staining in the most distal mitotic region (Figure 2B) and in germ cells entering meiotic diplotene in the proximal germ line (not shown). Almost all GLS-1 granules overlap with P granules, although GLS-1 intensities varied with respect to PGL-1 and GLH-2 intensities among P granules. This may reflect a selective enrichment of GLS-1 in P granules (Figure 2B). The ubiquitous GLS-1 expression overlaps the ubiquitous expression of GLD-3, yet GLS-1 and GLD-3 differ in their expression pattern during early and late meiotic prophase I (Figure 2A). As a specificity control, we stained germ lines extruded from *gls-1* mutant animals (Figure 2C). No immunoreactivity was observed with the monoclonal GLS-1 antibody, although GLD-3 (Figure 2C) and PGL-1 (not shown) were readily detected. We conclude that GLS-1 expression and subcellular localization in the germ line is dynamic and largely overlaps with GLD-3. GLS-1 is present in oocytes, which suggests a possible maternal role for *gls-1*.

In the early embryo, cytoplasmic GLS-1 is present in all cells, yet becomes gradually restricted to the germ cell lineage, and enriches in P granules (Figure 2D, data not shown). In early embryos we observed many GLS-1 positive particles that failed to stain strongly with P granule markers, yet stained readily for GLD-3 (Figure 2D, arrow). Granular GLS-1 and GLD-3 staining progressively overlapped with P granules during embryonic development and reached its maximum in the P4 germ cell precursor (data not shown). GLS-1 expression persisted throughout embryogenesis on P granules (data not shown). No GLS-1 was detected in embryos derived from *gls-1(ef8)* mothers (Figure 2D, bottom row). Interestingly, GLD-3 localization to P granules was still observed in these embryos (Figure 2D, compare GLD-3 and GLH-2 images), thus GLS-1 is not required for GLD-3 localization to P granules. Taken together, GLS-1 is dynamically expressed in the embryo and is a component of P granules in germ cell precursors. GLS-1 co-localizes with GLD-3 in the early embryo and both are enriched in germplasm granules that contain very little or no PGL-1 or GLH-2.

### GLS-1 Interacts Specifically with GLD-3

**Figure 3:**
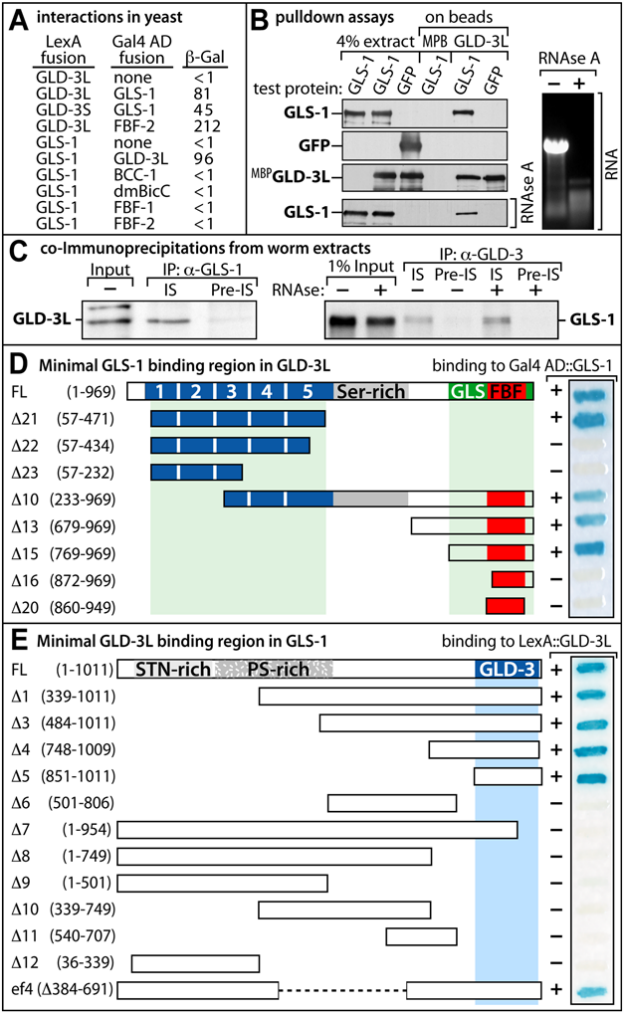
GLS-1 Binds GLD-3 Specifically. (A) Protein interactions as assayed in the yeast 2-hybrid system. β-galactosidase activities of yeast cells co-expressing protein pairs fused to the LexA DNA binding domain (DB) and the Gal4 transcriptional activation domain (AD) are given in arbitrary units. (B) Binding of GLS-1, GFP and GLD-3 co-produced in insect cells. Protein pulldowns with either MBP-GLD-3L or bacterially expressed MBP. Extracts were either RNAse A or mock incubated to demonstrate a non-RNA mediated interaction (bottom panel). The effectiveness of RNA digestion was monitored on an agarose gel (right panel). (C) Whole worm extracts were incubated with anti-GLS-1, anti-GLD-3L immune sera (IS) or the corresponding pre-immune serum (Pre-IS). Precipitated material was detected with the reciprocal antibodies. A prior treatment of the extract with (+) or without (−) RNase A treatment was monitored for efficiency on an agarose gel in the case of the GLD-3-coIP (not shown). (D) Identification of the minimal GLS-1 binding domain in GLD-3L. The strength of interaction with GLS-1 is rated according to the blue color of a β-galactosidase substrate (+ and − represent strong and weak binding, respectively). FL, full-length. (E) Identification of the minimal GLD-3 binding domain in GLS-1. The strength of interaction with GLD-3L on a filter assay is given and labeled as in (D).

To investigate the specificity of the GLS-1/GLD-3 interaction we performed further tests. GLS-1 interacted positively in yeast with both GLD-3 isoforms but not with other Bicaudal-C orthologs from *C. elegans* or *Drosophila*, e.g. *ce*BCC-1 or *dm*Bic-C. No interaction was observed with the GLD-3 interactors FBF-1 or FBF-2 (Figure 3A).

Next, we demonstrated a direct physical interaction between GLS-1 and GLD-3L with proteins expressed in insect cells (Figure 3B). All proteins carried a C-terminal His-tag and GLD-3L carried in addition an N-terminal Maltose Binding affinity tag (MBP). GLS-1 or GFP were co-expressed with GLD-3L by co-infection with baculoviruses encoding the individual fusion proteins. GLD-3L and associated proteins were captured on amylose resin and bead-bound proteins were analyzed by immunoblotting. GLS-1, but not GFP, was specifically pulled down by GLD-3L and not by MBP-coated beads alone (Figure 3B, lanes 4–6). To assess if the interactions were RNA mediated, we treated the extracts with RNAse A prior to the co-purification. The GLS-1 interaction with MBP::GLD-3L was still observed (Figure 3B).

In addition, we confirmed the existence of an in vivo GLS-1/GLD-3 complex by co-purifying each protein from wild-type worm extracts using protein-specific antibodies coupled to agarose beads (Figure 3C). Regardless of RNAse A treatment, GLD-3 and GLS-1 were strongly enriched in the anti-GLS-1 or anti-GLD-3 immunoprecipitate, respectively, but not in the IgG controls (Figure 3C).

Finally, we used protein truncations to map the interaction domain between GLD-3 and GLS-1 in yeast 2-hybrid tests. We detected two interaction surfaces within GLD-3L; an N-terminal region covering all KH domains and a C-terminal region containing the minimal FBF-binding region (Figure 3D). We mapped the GLD-3 interaction surface of GLS-1 to a single region of 160 amino acids at the very C-terminus (Figure 3E). Taken together, our results demonstrate a physical interaction between GLS-1 and GLD-3, mediated by specific regions within the proteins.

### *gls-1* Is Required for Germline Sex Determination and Germline Survival

To investigate the in vivo roles of *gls-1* we first reduced its gene function by RNA-mediated interference (RNAi) and observed 23% sterile progeny (n = 761) (Table 1). Nomarski analysis revealed animals with empty gonads (17%), and germ lines without oocytes (4%) or oogenesis defects (2%). Occasionally (<2% each), body shape defects, vulva defects, or embryos that failed to hatch were observed. In addition, less than 2% of the progeny contained a smaller but wild-type patterned germ line, indicative of proliferation defects. The missing germ cell phenotype reminded us of the germline survival (Gls) defect of *gld-3(RNAi)* progeny and suggested a shared function between GLS-1 and GLD-3.

**Table 1:** *gls-1* Phenotypic Defects.

	M+Z- (F1)^1^		M−Z- (F2)^1^		
Genotype 2	Sterile 3	Sperm (n) 4	Emb 5	Gls 6	n^7^
Wild-type	0%	∼340 (13)	no	0%	100
*gls-1(RNAi)*	23%	n.d.^8^	yes	16%	761
*gls-1(ef4)*	<2%	∼536 (9)	yes	61%	1456
*gls-1(ef8)*	<2%	∼548 (9)	yes	50%	1191
*gls-1(ef4)/nDf23*	100%	n.d.^8^	yes	n.a.^9^	28.
*gls-1(ef8)/nDf23*	100%	∼560 (6)	yes	n.a	41.
*+/nDf23* 10	<0%	∼468 (8)	n.a	n.a	>200
*gld-3(RNAi)* 11	86%	n.d.^8^	yes	80%	966
*gld-4(RNAi)* 12	80%	n.d.^8^	yes	20%	962

1 maternal (M) and zygotic (Z) defects in F1 or F2 progeny of mutant mothers; RNAi numbers were sorted accordingly into these categories.

2 hermaphrodite animals analyzed at 20°C.

3 no embryos present.

4 total number of sperm per germ line arms scored (n).

5 embryonic lethality observed.

6 germline survival defect as a percentage of all analyzed progeny.

7 total germ line arms scored.

8 not determined.

9 not applicable.

10 animals are also heterozygote for *unc-13 lin-11*.

11 taken from [5].

12 hermaphrodite animals analyzed at 25°C.

To characterize *gls-1* functions in greater detail we isolated two chromosomal deletion mutants, *gls-1(ef4)*, a central in-frame deletion, and *gls-1(ef8)*, a central out-of-frame deletion (Figure 1B, see Materials and methods). Consistent with the truncated mRNA coding potentials, we detect robust expression of GLS-1^ef4^ protein by immunoblotting with a C-terminal rabbit anti-GLS-1 antibody and *gls-1(ef8)* protein extract serves as a specificity control (Figure 1E). Genetic evidence presented later suggests that both *gls-1* mutations behave as reduction-of-function alleles at lower temperatures and as genetic null alleles at elevated temperatures.

For our overall phenotypic analysis we separated zygotic (Z) and maternal (M) functions of *gls-1* by scoring adult mutants that were either the F1 progeny (*gls-1* M+Z-) of heterozygote *gls-1*/+ mothers, or their F2 progeny (*gls-1* M−Z-) (Table 1). We discovered that *gls-1* M+Z- animals appeared to contain excess sperm (∼94%, n = 107) and occasionally produced sperm exclusively (∼2%, n = 107); a detailed sperm count is given in Table 1. Furthermore, we observed germ lines that switched to sperm production in the distal region adjacent to more proximal germ cells undergoing oogenesis (∼4%, n = 107). A lack of both maternal and zygotic *gls-1* activity (M−Z-), caused animals to display the germline survival defect at a high frequency, hence its name. Somatic defects that appeared phenotypically similar to the RNAi defects were observed at a low frequency, but were not further pursued. In summary, we conclude that *gls-1* has a zygotic role in promoting the switch to oogenesis and a maternal role in ensuring germline survival.

### *gls-1* Mutant Germ Lines Display Severe Oogenesis Defects

**Figure 4:**
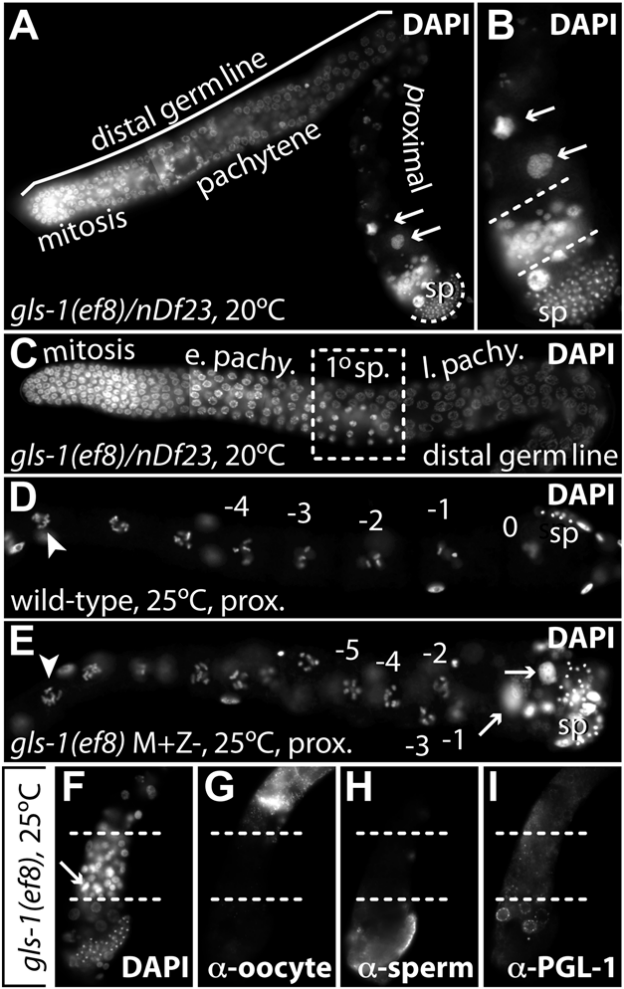
Oogenesis Defects in *gls-1* Mutants. (A, B) Extruded germ line of a *gls-1/nDf23* hemizygote animal raised at 20°C stained for nuclear morphology. (B) Enlargement of proximal end in A. sp, mature sperm; arrows, abnormal endoreduplicating oocyte nuclei. Dashed lines mark a group of cells with small decondensed nuclei of various sizes that do not match typical meiotic germ cell nuclei. (C) Distal germ line of a *gls-1/nDf23* hemizygote animal. e. pachy., early pachytene. l. pachy., late pachytene. 1°sp, primary spermatocytes in late prophase I. (D, E) Proximal germ lines of wild-type and a *gls-1* homozygote progeny from a *gls-1*/+ mother raised at 25°C in the last larval stage. Distal to the left, proximal (prox.) to the right. Numbers denote oocyte nuclei. Note, *gls-1(ef8)* germ lines have multiple rows of oocytes. Arrowhead, diplotene oocyte. Other additional labels as in A. (F–I) Most proximal end of a *gls-1(ef8)* homozygous germ line from the progeny of a *gls-1*/+ mother raised at 25°C in the last larval stage. Small decondensed nuclei of various sizes and showing mitotic behavior are located between dashed white lines. Arrow, metaphase plate. (F) DAPI staining. (G) anti-RME-2 (H) SP56 staining (I) P granules.

To determine the phenotypes of strong loss-of-function *gls-1* mutants we generated animals hemizygote for *gls-1* M+Z- by placing each *gls-1* mutation in *trans* to the genomic deficiency *nDf23* (Figure 1A). In contrast to +/*nDf23* animals, all collected adult hemizygotes were fully sterile and produced no living progeny (Table 1, Figure 4A); occasionally they contained a dead embryo in their uterus. The sterility was due to defects in oogenesis, as sperm were present in all animals and homozygous males sire normal progeny; on average sperm were present in excess similarly to the homozygous *gls-1* mutants (Table 1). In rare cases, *gls-1*/*nDf23* hemizygotes contained either fully masculinized germ lines with no signs of oogenesis, or their distal germ cells reverted from oocyte to sperm production suggesting that the switch to oogenesis is not maintained in adult worms (Figure 4C). In young adult hermaphrodites these germ cells are first immunoreactive with the sperm specific differentiation marker SP56 and they subsequently mature in older animals into sperm (not shown). However, the number of proximal sperm produced is very similar between *gls-1* homozygotes and *gls-1/nDf23* hemizygotes (Table 1), suggesting that both *gls-1* alleles, while perhaps not nulls, are at least strong loss-of-functions with respect to the sperm-to-oocyte switch.

The oogenesis defects in animals hemizygous for *gls-1* are complex. Most germ lines maintained the oogenic fate, yet lack the linear array of large oocytes arrested in diakinesis that is found in wild-type (compare Figure 4A and 4D). Rather, oogenic cells remained small and were either arrested in pachytene or underwent abnormal meiotic prophase progression and failed to arrest in diakinesis, often resulting in endoreduplicating oocyte nucei (Figure 4A,B). Furthermore, we observed in the most proximal germ line aberrant nuclei positioned between mature sperm and oocytes that showed no signs of clear gamete differentiation markers (Figure 4A,B and F–I). In addition, we noticed an abnormal oocyte membrane organization (Figure 4G), which was also seen in anti-actin and anti-anillin (ANI-2) antibody stainings (not shown). We conclude that *gls-1* activity is required for proper oocyte differentiation and oogenic meiotic arrest.

Interestingly, we can induce these oogenesis defects also in our single *gls-1* homozygous mutants by shifting mid-L4 larvae to 25°C. The displayed germline defects are very similar to the phenotypes of *gls-1/nDf23* hemizygotes at 20°C and all analyzed animals were sterile (n>100) (compare Figure 4A and 4E). We also examined the phenotype of *gls-1(ef4)/nDf23* animals at 25°C. No further enhancement of the germ line phenotypes were seen (25/25) and *+/nDf23* animals were fertile and appeared wild-type (39/40). We conclude that, for oogenesis defects, both *gls-1* mutations behave as genetic null alleles at elevated temperatures.

### *gls-1* Acts Redundantly with *fbf-1* and *nos-3* to Promote the Female Fate of Germ Cells

To investigate the relationships between *gls-1* and *gld-3* and to understand their molecular interactions we first focused on their seemingly opposing roles in germline sex determination. Zygotic *gld-3* is required for the sperm fate; *gld-3(0)* mutants display a feminized germ line in both sexes [5],[20]. In contrast, *gls-1* mutations masculinized the hermaphrodite germ line (Table 1). Other known regulators of the sperm-to-oocyte switch include three PUF proteins (FBF-1, FBF-2 and PUF-8) and NOS-3. The former are required for suppressing the sperm fate by translationally repressing several sperm promoting genes of the sex determination cascade and the latter reinforces FBF activity at the level of *fem-3* mRNA regulation [21],[22]. Interestingly, each single mutant produces sperm and oocytes, similar to *gls-1*. However, PUF double mutants produce only sperm, exemplifying redundant controls in promoting oogenesis.

**Figure 5:**
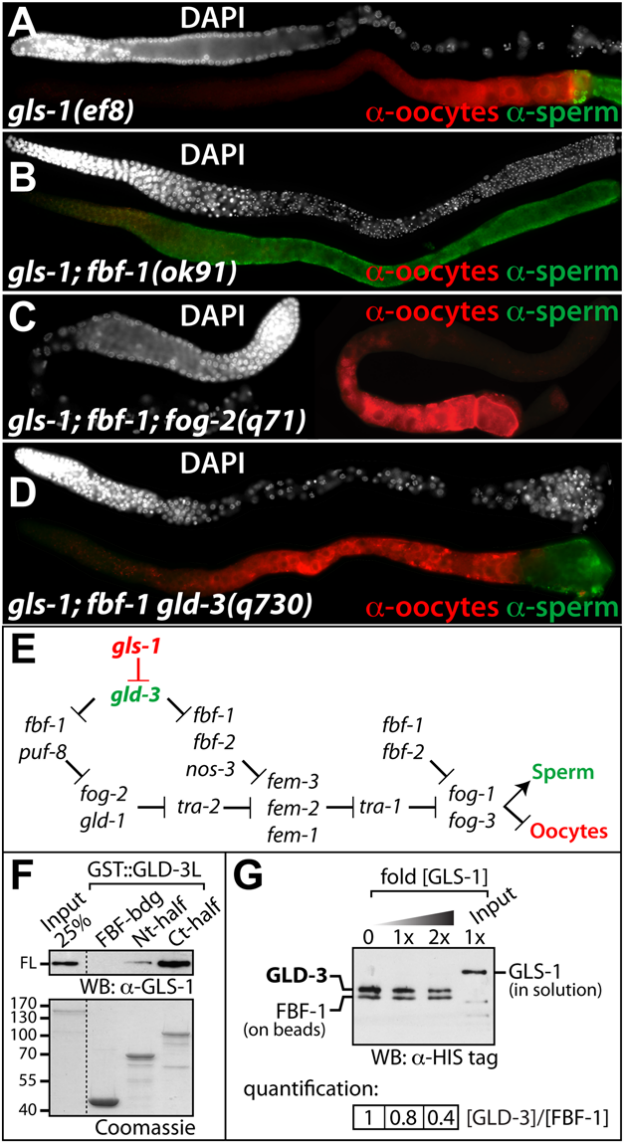
GLS-1 Regulates the Sperm-to-Oocyte Switch. (A–D) Immunofluorescence images of extruded germ lines stained for sperm (SP56, green), oocytes (anti-RME-2, red) and nuclear morphology (DAPI, white). (E) Summary of the genetic cascade regulating the sperm-to-oocyte switch in the hermaphrodite germ line. Previously established genes (in black) taken from [11] and new players are indicated in color. (F) GLS-1(FL) has a stronger affinity for the unique C-terminal half of GLD-3L in GST-pulldown assays. (Top) GLS-1 visualized with anti-His antibodies. (Bottom) Purified full-length GLS-1^6xHis^ and GLD-3 fragments fused to GST. Nt-half, covers all KH domains. Ct-half, covers the unique part of GLD-3L. (G) Competition assay for GLD-3L and GLS-1 on bead bound FBF. All proteins are purified from insect cells and carry a C-terminal His tag. FBF is immobilized on beads. GLD-3L was first bound to FBF and subsequently displaced by the addition of increasing amounts of free GLS-1. The decrease of GLD-3L on beads was quantified.

To understand the role of *gls-1* in sex determination and to test for a likely redundancy with *fbf*, we combined *gls-1* with other sperm-to-oocyte switch defective genes and generated double mutants. We analyzed the germ lines with Nomarski and immunofluorescence microscopy to unambiguously identify the gamete fate in young adult hermaphrodites. We discovered an essential role for GLS-1 in the switch to oogenesis when FBF-1 is not expressed (Table 2); *gls-1*; *fbf-1* double mutants are fully sterile and produced only sperm and no oocytes (Table 2; Figure 5B). In contrast, compromising *gls-1* activity in a *fbf-2* or *puf-8* mutant background does not cause a sperm only (Mog) phenotype; sperm and oocytes were always present (Table 2). The synthetic Mog phenotype in double mutants was not enhanced or changed at elevated temperatures, which is consistent with *gls-1(ef8)* being a strong loss-of-function with respect to the sperm-to-oocyte switch. When we simultaneously eliminated *nos-3* and *gls-1* function, we observed a rather complex phenotype; germ cells either failed to adopt the oocyte fate entirely (∼20%) or were not able to maintain the oogenic fate and produced sperm cells in the distal arm at a much higher frequency (∼20%) than *gls-1(ef8)* by itself (1%) (Table 2). However, the remaining 60% of *gls-1*; *nos-3* germ lines switched to and maintained oogenesis. In summary, we find that *gls-1* works genetically in parallel, or together, with *fbf-1* and *nos-3* to promote the sperm-to-oocyte switch in hermaphrodites.

**Table 2:** Sperm-to-Oocyte Switch Defects in *gls-1* Mutants.

Genotype 1	Sp 2+Oo 2	Sp 2 only	Oo 2 only	n^3^
Wild-type	100%	0%	0%	>200
*gls-1(ef8)*	99%	1%	0%	83
*fbf-1(ok91)* 4	99%	1%	0%	570
*gls-1(ef8); fbf-1(ok91)*	0%	100%	0%	80
*fbf-2(q738)* 4	99%	0%	1%	2201
*gls-1(ef8); fbf-2(q738)*	100%	0%	0%	25
*nos-3(q650)* 5	>99%	<1%	0%	2000
*gls-1(ef8); nos-3(q650)* 6	59%	41%^6^	0%	44
*puf-8(q725)* 7	97%	3%	0%	1952
*gls-1(ef8); puf-8(q725)*	100%	0%	0%	149
*gls-1; fbf-1; fem-1(RNAi)* 8	42%	58%	8%	26
*gls-1; fbf-1; fem-2(RNAi)* 8	50%	42%	8%	24
*gls-1; fbf-1; fem-3(RNAi)* 8	0%	4%	96%	75
*fog-1(RNAi) gls-1; fbf-1*	0%	1%	99%	94
*fog-2(q71)* 9	0%	0%	100%	44
*gls-1; fbf-1; fog-2(q71)* 9	18%	0%	82%	39
*gld-3(q730)* 10	87%	0%	13%	110
*gls-1(ef8); gld-3(q730)* 11	n.a	n.a	n.a	>100
*fbf-1(ok91) gld-3(q730)* 12	100%	0%	0%	519
*gls-1; fbf-1 gld-3(q730)* 13	97%	2%	1%	105

1 animals grown at 20°C.

2 Sp, sperm; Oo, oocytes.

3 total number of germ lines scored.

4 Taken from [27].

5 Taken from [13].

6 The number includes >20% fully Mog germ lines and >20% germ lines that return to spermatogenesis after producing oocytes.

7 Taken from [22].

8 from *gls-1(ef8)/ccIs4532; fbf-1(ok91)/mIn1g)* mothers.

9 from *fog(q71) unc-51(e1189)/+* mothers (see strains).

10 Taken from [5].

11 animals do not produce gametes.

12 the amount of sperm is similar to *fbf-1* single mutants, which is elevated compared to wild-type [26].

13 animals have no living progeny.

### *gls-1* Acts Upstream of All Known Sperm-Promoting Factors in the Germ Line

To place the action of *gls-1* and *fbf-1* into the sex determination pathway we performed genetic epistasis experiments with key spermatogenesis promoting genes. We either generated true triple mutants or relied on feeding RNAi experiments to generate “triple mutant” phenotypes (Table 2). Interestingly, the synthetic Mog phenotype depended on the activity of each known *fem* and *fog* gene. All “triple mutant” germ lines produced either oocytes only or oocytes in addition to sperm. The mixture of sexual fates might be due to an incomplete penetrance caused by RNAi; the wild-type animals treated in parallel were fully feminized to a similar percentage as the *gls-1*; *fbf-1* mutants (not shown). The *gls-1*; *fbf-1*; *fog-2* triple mutant also displayed a mixture of cell fates, yet we observed mostly feminized germ lines (82%, Table 2; Figure 5C). Taken together, we provide evidence that *gls-1* is upstream of all known spermatogenesis-promoting genes, similar to the activity of *fbf-1*.

To determine if this masculinization defect is due to the activity of *gld-3*, we generated the *gls-1(ef8); fbf-1(ok91) gld-3(q730)* triple mutant. As expected, we were able to restore the oocyte fate in *gls-1; fbf-1* germ lines by eliminating *gld-3*; almost all triple mutant germ lines displayed a sperm and oocyte pattern (97%, Table 2; Figure 5D). Therefore, we conclude that the synthetic Mog phenotype of *gls-1; fbf-1* germ lines depends on excess *gld-3* activity (summarized in Figure 5E).

### GLS-1 Competes with FBF for GLD-3 Binding

Given the known molecular interactions of GLS-1 and GLD-3 (Figure 3), the simplest interpretation is that GLS-1 is a modulator of the sperm-to-oocyte switch by acting antagonistically to GLD-3 and in parallel to FBF. In this scenario, the switch to oogenesis is promoted by relieving FBF from GLD-3L inhibition through GLS-1 binding. Support for this model comes from our in vitro observations that the C-terminal half of GLD-3L binds consistently with higher affinity to GLS-1 than the N-terminal half (Figure 5F). To test the proposed antagonistic model further we employed a competition experiment. FBF-1 was immobilized on beads and subsequently loaded with GLD-3L. Afterwards, the FBF-1/GLD-3L complex was challenged with increasing amounts of free GLS-1. GLS-1 and dissociated GLD-3L was washed away. We observed that the GLD-3 amount bound to FBF decreased with higher GLS-1 concentrations present, while FBF-1 remained stably bound to the beads (Figure 5G). Together, this data indicates that GLS-1 protein can recruit FBF-1 from the interaction with GLD-3. Therefore, GLS-1 may liberate FBF-1 for translational repression of spermatogenic factors.

### Maternal *gls-1* Activity Prevents Progressive Germ Cell Degeneration

**Figure 6:**
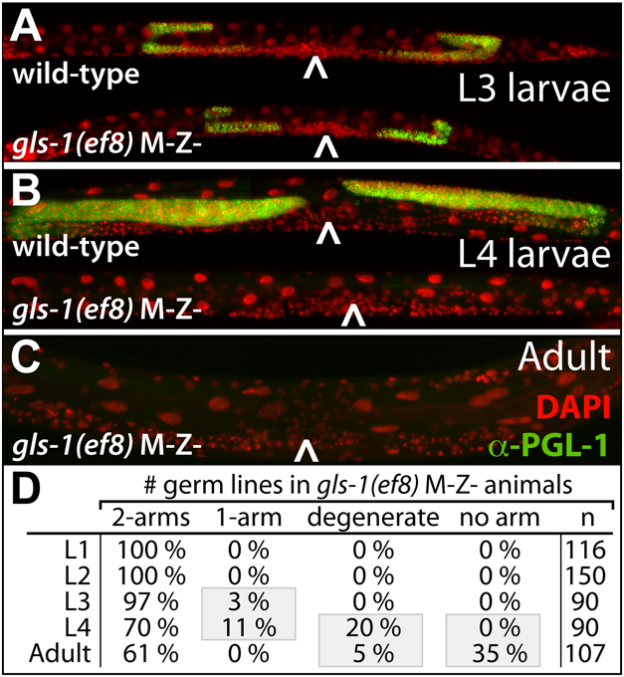
The Maternal Germline Survival Defect in *gls-1* Mutants. (A–C) Whole mount staining of indicated genotypes and staged animals. Caret, position of vulva. False colored DAPI (red) and P granules (green). (D) Summary of the Gls phenotype present in the *gls-1(ef8)* F2 progeny. A degenerate arm is classified as a gonadal arm that contains no more than 20 germ cells.

Next, we addressed how GLS-1 influences GLD-3 activity to promote germline survival. The adult wild-type hermaphrodite contains two U-shaped gonadal arms filled with ∼1000 germ cells [23]. By contrast, *gls-1(RNAi)* hermaphrodites and homozygote *gls-1* M−Z- adult animals often contain an empty somatic gonad with no apparent germ cells (Table 1), resembling the maternal *gld-3* mutant Gls phenotype. Hence, we characterized the genesis of the *gls-1* M−Z- adult sterility during postembryonic development by Nomarski microscopy (not shown) and visualized germ cells by DAPI and anti-P granule staining (Figure 6A–C).

During the first two larval stages germ cell development of *gls-1(ef8)* M−Z- hermaphrodites is similar to wild-type; L1 larvae hatch with two progenitor germ cells and contain at early L2 an approximately wild-type number of germ cells. After the formation of an anterior and posterior gonadal arm in late L2/early L3, both germ lines were clearly present in *gls-1(ef8)* M−Z- animals. A subtle, but apparent, germline size difference to the wild-type was visible at the L3 stage (Figure 6A,D) and became more pronounced as development progressed. Roughly one third of *gls-1(ef8)* M−Z- L4 animals displayed evident germ line loss and contained very few or no PGL-1 positive germ cells (Figure 6B,D). The phenotype was exacerbated in the adult; 40% *gls-1(ef8)* M−Z- animals had either lost the entire germ line or it was strongly degenerated and reduced to a few unhealthy looking germ cells (Figure 6C,D). Interestingly, the germ cell loss phenotype was still present in *gls-1(ef8); ced-4(n1162)* animals, where the canonical apoptotic pathway is blocked (not shown). We conclude that in embryos lacking maternal *gls-1* function, germ cells are correctly specified, initiate proliferation and are subsequently lost as a result of progressive degeneration, which begins to be seen as early as the L3 larval stage (Figure 6D). This phenotype mimics the germline survival defect of maternally depleted *gld-3* animals and is consistent with a shared and common function for maternal *gls-1* and *gld-3*. We recently identified the novel poly(A) polymerase GLD-4 as an interactor of GLS-1 [24]. Interestingly, RNAi knockdown of zygotic and maternal *gld-4* by injection results in progeny with a high penetrance Gls phenotype at elevated temperatures (Table 1), suggesting that all three proteins may form a maternal complex to promote germline survival.

### The GLD-3/GLS-1 Complex Ensures Germline Survival

**Figure 7:**
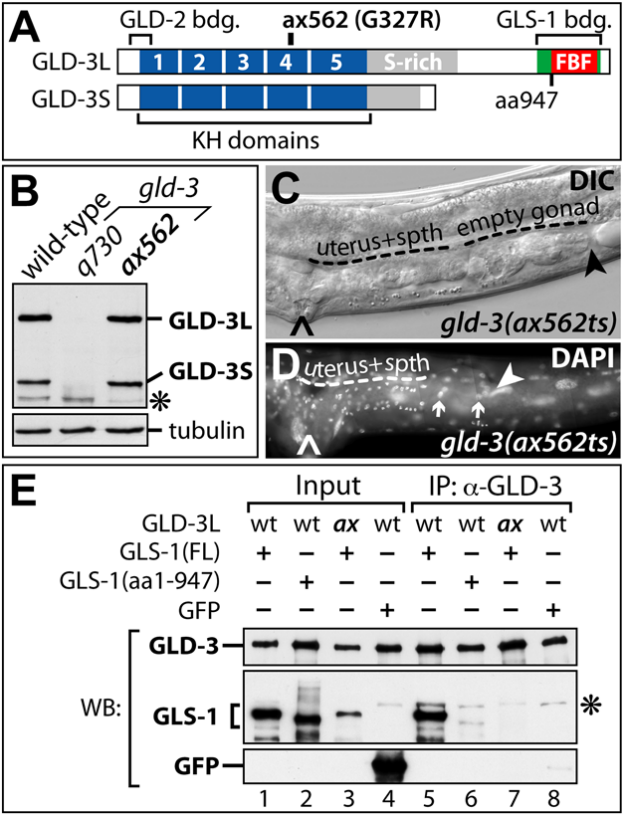
Germline Survival Depends on a GLS-1/GLD-3 Complex. (A) Schematic diagram of GLD-3 domains and the position of the *gld-3(ax562ts)* missense mutation. (B) Immunoblot for both GLD-3 isoforms. The *gld-3(q730)* allele is protein null. (C–D) Adult *gld-3(ax562ts)* hermaphrodites grown at restrictive temperature after being shifted during early embryogenesis (<100 cell stage). (C) Nomarski image. (D) whole mount DAPI image. No germ cells are visible in the gonadal tissue. spth, spermatheca. Arrows, sheath cell nuclei. Arrowhead, distal end. Caret, vulva. (E) GLD-3^ax562^ missense mutation impairs GLS-1/GLD-3L complex formation. Proteins co-expressed in insect cells. Wild-type(wt) or ax562(ax) MBP::GLD-3L isoforms are precipitated by an anti-GLD-3 N-terminal antibody whose epitope is outside the interaction domains. Asterisk, high molecular weight background band.

To test the hypothesis that the common biological role of maternal GLS-1 and GLD-3 in germline survival is linked to their physical interaction, we utilized a specific temperature-sensitive *gld-3* mutation. The *gld-3(ax562)* allele contains a missense mutation that leads to a glycine-to-arginine (G/R) substitution in the fourth KH domain of GLD-3S and GLD-3L (Figure 7A). Homozygote *gld-3(ax562)* animals are fertile at permissive temperature and contain a superficially normal germ line. Strikingly, when shifted to the restrictive temperature as early embryos, these animals display the germline survival defect (25%, n = 130, Figure 7C,D), even though both GLD-3 isoforms are expressed to wild-type levels (Figure 7B).

As this phenotype is similar to *gls-1* removal, we asked if the G/R change would affect the GLS-1/GLD-3 interaction. Hence, we co-expressed both proteins in insect cells and consistently found that wild-type GLD-3 protein, but not GLD-3^ax562^, was able to associate with GLS-1 in co-immunoprecipitation experiments (Figure 7E). A C-terminal truncation of GLS-1 missing the GLD-3-binding region served as a negative control. These results were similar to our directed yeast 2-hybrid binding-tests (not shown), and do not depend on the temperature that cells were grown at (see Text S1). Interestingly, GLD-3^ax562^ bound efficiently to the positive control, GLD-2, in insect cells and yeast (not shown). We conclude that the *gld-3(ax562)* point mutation specifically abrogates the interaction with GLS-1. Taken together, our analysis suggests that the maternal GLS-1/GLD-3^ax562^ complex is in vivo temperature sensitive and that upon dissociation of the complex, germ cells fail to survive into adulthood.

## Discussion

In this study we addressed how the conserved RNA regulatory core machinery, i.e. GLD-3/Bic-C, FBF/Pumilio and NOS-3/Nanos, that executes cell fate decisions, is regulated to implement organism-specific developmental germline programs. Our analysis focuses on the isolation and characterization of the novel P granule component, GLS-1, a protein important for the sperm-to-oocyte switch, proper oogenesis, embryogenesis and germline survival. We find that GLS-1 may directly manipulate the output of the RNA regulatory network by modulating GLD-3 availability and activity. Our data provides a molecular framework for how a single molecule may have evolved as a novel germplasm component to integrate germ cell viability with germ cell differentiation.

**Figure 8:**
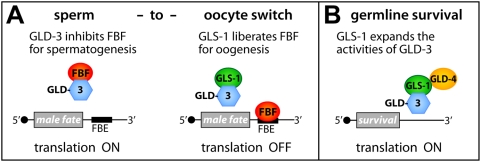
Models of How GLS-1 May Modulate GLD-3 in Cell Fate Decisions. (A) GLS-1 antagonizes GLD-3 in the sperm-to-oocyte switch to allow translational repression of FBF-binding elements (FBE) containing mRNAs encoding male fate factors. (B) GLS-1 synergizes with GLD-3 for germline survival and may expand its functions by bridging to GLD-4 to translationally activate mRNAs encoding survival factors; presumably by poly(A) tail elongation.

The formation of a GLS-1/GLD-3 protein complex is redeployed multiple times throughout germline development (Figure 8). The formation of the zygotic GLS-1/GLD-3 complex promotes the oogenic cell fate by liberating the translational repressor FBF/Pumilio to turn off sperm promoting factors. In this scenario, GLS-1 acts as a molecular mimic of FBF/Pumilio. Possibly, this interaction may also redirect the putative RNA-binding protein GLD-3 to actively promote the oocyte fate and oogenesis *per se*. The formation of a maternal GLS-1/GLD-3 complex is required to promote germline survival during postembryonic germline development. In the latter decision, GLS-1 putatively transforms GLD-3 into a translational activator most likely by recruiting additional factors, such as the cytoplasmic poly(A) polymerase GLD-4 [24].

### GLS-1 Modulates GLD-3L to Enhance the Switch to the Oocyte Fate

The sperm-to-oocyte decision is remarkably complex and serves as a paradigm for post-transcriptional control mechanisms. Translational repression of *fem-3* mRNA is essential for female germ cells to acquire the oocyte fate. FEM-3 is a novel protein and forces male fate development [25],[26]. Translationally inactive *fem-3* mRNA is repressed by two conserved PUF proteins (FBF-1 and FBF-2) and NOS-3, which are thought to form an RNP complex [13],[14]. To allow transient spermatogenesis in the hermaphrodite and continuous sperm production in the male, *fbf* activity is antagonized by *gld-3*. GLD-3 regulates the timing of the hermaphroditic sperm-to-oocyte switch, i.e. the numbers of sperm produced, and maintains the sperm fate in the male [5]. Molecularly, GLD-3L promotes the sperm fate by directly contacting the RNA-binding domain of FBF. As a consequence, FBF's affinity to its cognate target sequence in *fem-3* mRNA is weakened; this promotes *fem-3* mRNA translation and delays the onset of oogenesis [5].

How is FBF activated to overcome GLD-3L inhibition to execute the sperm-to-oocyte switch at the correct time? Furthermore, how is this repression maintained in the aging hermaphrodite for continuous oocyte production? Our work on GLS-1 provides support for a model in which FBF is permanently liberated from GLD-3L repression by the formation of a GLS-1/GLD-3L complex (Figure 8A). This interaction requires a region within GLD-3L that includes the entire minimal FBF-binding site. As the GLD-3 interaction sites in FBF and GLS-1 differ in their primary sequence, we regard GLS-1 as a molecular mimic, and not necessarily a structural mimic, of FBF. Our molecular model is based on the following findings: GLS-1 is expressed in the L4 gonad (data not shown) when oocytes are specified. *gls-1* hermaphrodite mutants produce almost twice as many sperm than wild-type, which is similar to *fbf-1* single mutants [27]. A synergistic loss of *gls-1* and *fbf-1* prevents the switch to oogenesis entirely and these masculinized germ lines depend on *gld-3* activity. Furthermore, our in vitro protein binding competition results are consistent with the conclusion that GLS-1 performs its function by directly interfering with the GLD-3L/FBF interaction.

FBF-1 is the only RNA-binding protein that is known to be involved in all well characterized translational regulations that affect the sperm-to-oocyte switch (see Figure 5E). For example, *fbf-1*, but not *fbf-2*, activity is required to repress *fog-2*, a gene more upstream in the sex determination pathway than *fem-3*. However, FBF-1 requires the help of PUF-8 to repress *fog-2* mRNA [22]. Similarly, FBF-1 depends on FBF-2, and presumably NOS-3, to fully repress *fem-3* mRNA [13],[14]. Our data are also in good agreement with a more central role attributed to *fbf-1*, rather than *fbf-2*, *nos-3* and *puf-8*, in the sperm-to-oocyte switch (see Figure 5E). *gls-1; fbf-1* double mutants fail to produce oocytes and produce sperm only, whereas *gls-1; fbf-2* and *gls-1; puf-8* double mutants produce sperm and oocytes. Consistent with our model of FBF liberation from GLD-3L repression by GLS-1, we find that *gls-1* also acts upstream of *fog-2* and requires *gld-3* activity. These findings suggest that FBF-1 regulation is tightly linked to GLS-1/GLD-3 complex activity and that all FBF-1 spermatogenetic target mRNAs may require the assistance of GLS-1 for translational repression. In summary, it seems likely that FBF-1 and GLD-3 provide the core of the switch machinery and that GLS-1, NOS-3/Nanos, and the other PUF proteins are accessory modulators for the sperm-to-oocyte switch. Yet, it remains an open question, how GLD-3L handover from FBF-1 to GLS-1 is regulated and executed in a developmental fashion. GLS-1 indirectly promotes the switch by releasing FBF to inhibit sperm production. Yet, it remains to be determined if GLD-3 sequestration from the GLD-3L/FBF complex may also be a way to modulate the roles of GLD-3 activity, to directly promote oogenesis through the translational regulation of yet unknown oocyte-promoting mRNAs. This scenario seems likely given their common maternal activities.

### The Maternal GLS-1/GLD-3 Complex Is Critical for Germline Survival

We find that a reduction of maternal *gls-1* or *gld-3* activity in the early embryo, results in a common phenotype, i.e. the germline survival defect. A hallmark of the Gls defect is that germ cells are correctly specified in the early larvae, proliferate until the L3 stage and degenerate rather than differentiate in later development. This form of germ cell death appears to a large extent independent of CED-3 or CED-4-mediated apoptosis [5, this work]. Although a more detailed pathological analysis of this phenomenon is missing, a molecular framework for how germline survival may be achieved becomes apparent. We propose that the formation of a larger GLS-1/GLD-3 RNP complex positively influences the translational activity of distinct mRNA(s), which encodes a germline survival factor (Figure 8B). Alternatively, it may repress a germline death factor. The recent discovery of the cytoplasmic poly(A) polymerase GLD-4, which physically binds to GLS-1, suggests the involvement of poly(A) tail length control [24].

Our model is strongly supported by our analysis of the GLS-1 interaction-deficient *gld-3(ax562ts)* mutation. At restrictive temperature *gld-3(ax562)* animals produce both predominant GLD-3 isoforms to wild-type levels but carry a single glycine-to-arginine (G/R) replacement in their fourth KH domain. The bulky, charged side chain of arginine is expected to have severe structural consequences on KH domain folds [28]. Consistent with this, we find that GLD-3^ax562^ does not form a complex with GLS-1 in vitro. Importantly, other known protein interactors of GLD-3, e.g. GLD-2, are not compromised in interacting with GLD-3^ax562^ and GLS-1 remains expressed in *gld-3(ax562)* embryos at restrictive temperature (Rybarska and Eckmann, unpublished results). Together this suggests that the G/R substitution disturbs GLD-3 folding locally to specifically inhibit GLS-1/GLD-3 complex in contrast to distorting global GLD-3 structure and compromising all GLD-3 functions.

We have considered the possibility that the mutation in the KH domain of the *gld-3(ax562)* allele might affect the RNA-binding capacities of GLD-3, as KH domains are known to serve as RNA-binding surfaces as well as protein interaction platforms [29]. However, we found the general RNA-binding affinity of GLD-3L^ax562^ in RNA homopolymer-binding assays unaffected when compared to GLD-3L^WT^ (Jedamzik and Eckmann, unpublished results). Therefore, the G/R substitution seems more likely to compromise the formation of a GLS-1/GLD-3 complex rather than interfering with interactions with mRNA targets. Unfortunately, no target mRNAs required for germline survival have been identified to date. Nevertheless, our working model invokes the formation of a larger GLS-1/GLD-3 RNP complex that positively regulates a distinct mRNA target(s) (Figure 8B). We speculate that this type of regulation might involve poly(A) tail metabolism as GLS-1 is able to bind and stimulate the poly(A) polymerase activity of GLD-4 [24]. In the current work we find that *gld-4(RNAi)* induces Gls animals at elevated temperatures. Hence, GLS-1 might recruit GLD-3 into a trimeric complex with GLD-4, and together they may be critical for germline survival.

Additional unknown components may influence the structure/stability and function of maternal GLS-1/GLD-3 RNP complexes in the early embryo. Analysis of the *gld-3(ax562)* temperature-sensitive Gls phenotype would support this. The GLS-1/GLD-3^ax562^ complex is only severely compromised in vivo at higher temperatures. In contrast, in vitro or in yeast the GLS-1/GLD-3^ax562^ complex does not form and we infer that other factors might stabilize the complex in vivo at permissive temperature. Considering the expression and co-localization of GLS-1 and GLD-3 in early embryos, we propose that germline survival is initiated, as a function of the maternal GLS-1/GLD-3 complex, during early embryogenesis rather than early postembryonic development. This is also consistent with the temperature sensitive period of *gld-3(ax562)*. Homozygote *gld-3(ax562)* animals produce adult animals without germ lines when shifted during early embryogenesis. Later shifts have a less detrimental effect on germ cell loss even when maintained at high temperatures (Rybarska and Eckmann, unpublished results).

In summary, GLS-1 is a master modulator of multiple GLD-3 functions throughout germline development. GLS-1 limits and extends GLD-3 availability by providing an interactive platform to bring in additional modulators to form new RNP complexes with varied activities. Proteins functionally analogous to GLS-1 are highly likely to exist in other organisms. Modulators of conserved RNPs are undoubtedly required to implement cell fate decisions during development across animals. As metazoan germ cells depend strongly on post-transcriptional regulatory mechanisms, these functional analogues may also be found in their germplasm granules.

## Materials and Methods

### Strains

Worms were handled according to standard procedures and grown at 20°C unless otherwise stated [30]. Strains used in this study: LGI: *gls-1(ef4)*, *gls-1(ef8)*, *nDf23/unc-13(e1091) lin-11(n566)*, *glp-4(bn4ts)*; LGII: *gld-3(q730), gld-3(ax562ts)*, *fbf-1(ok91)*; LGIII: *glp-1(q224ts)*, *ced-4(n1162)*; LGV *unc-51(e1189)*, *fog-2(q71)*; the wild-type strain was bristol N2. The *gls-1(ef4)* and *gls-1(ef8)* deletion mutants were generated in an EMS based deletion screen [13] and are described further in Text S1. Adult germ line phenotypes were scored 24 hrs past midL4.

### Antibody Production and Immunocytochemistry

Antibodies against the following proteins were used as described: anti-GLD-1 [31], anti-FOG-2 [32], SP56 [33], anti-RME-2 [34], anti-GLD-3 [20]; anti-PGL-1 [35], anti-GLH-2 [19]. A rabbit polyclonal antibody serum (C5C0) was generated against a GST::GLS-1 fusion comprising aa 249–576. Affinity purification was carried out using a maltose binding protein fusion of the identical GLS-1 piece immobilized on a HiTrap (Amersham) column and eluted at low pH. A monoclonal antibody was generated immunizing mice with a peptide corresponding to the very C-terminus of GLS-1 (aa 989–1011). The mouse anti-GLS-1 antibody (mo184C16) recognized in vitro produced GLS-1 protein (not shown) and is very specific for GLS-1 in staining experiments. However, we found that it is also more sensitive to paraformaldehyde (PFA) fixation and thus stains less prominently granules. For some P granule double labelling experiments we used an anti-PGL-1 peptide antiserum of guinea pigs that stained P granules very similar to published anti-PGL-1 antibodies [35]; the affinity purified antibody is specific as no signal was observed in *pgl-1(bn101)* animals. Immunofluorescence on extruded and 1% PFA fixed germ lines was carried out in solution as described [5]. Embryo (Figure 2D) and whole worm immunocytochemistry (Figure 6) with methanol/acetone fixation was described elsewhere [36]. Images were taken on a Zeiss Imager M1 equipped with an Axiocam MRm (Zeiss) and processed with AxioVision (Zeiss) and Photoshop CS3 (Adobe). For optical sections we either generated images on an Imager Z1 with an Apotome (Zeiss) or on a confocal microscope (LSM Meta510, Zeiss). Secondary antibodies were coupled to fluorochromes FITC, CY3 and CY5 (Jackson Laboratories).

### Protein Biochemistry and Immunoblotting

Recombinant GST fusions of GLD-3L fragments were produced in BL21(pRIL) *E. coli*. Recombinant C-terminally His-tagged protein fusions of GLS-1, GFP and GLD-3L (wt and ax562) were produced in SF+ insect cells with the help of baculoviruses, which were generated and tested for their protein expression levels in SF+ insect cells according to the manufacturers protocols (Invitrogen). Co-expression was performed by viral co-infection.

MBP::GLD-3L and GST::GLD-3 fragments were purified on amylose resin (NEB) and glutathione beads (Sigma), respectively. GLS-1^6His^ and derivatives were purified in SF+ lysis buffer [50 mM HEPES pH 7.5, 100 mM NaCl, 5% glycerol, 0.1% Triton X-100, 1 mM DTT, Protease inhibitor cocktail (Roche) and E64 (BioMol)] on Ni-NTA agarose beads (Qiagen), eluted with 250 mM Imidazole/50 mM HEPESpH 7.5/500 mM NaCl and dialysed against DB200 [20 mM Tris-Cl pH 7.5, 200 mM NaCl, 0.1% Triton X-100]. For the competition assay MBP-GLD-3^6His^ and MBP-FBF-1^6His^ were purified on amylose beads (NEB) in SF+ lysis buffer. The N-terminal MBP fusion part of GLD-3L^6His^ was cleaved off by PreScission protease treatment (GE Healthcare). Western Blot bands were quantified in Adobe Photoshop CS2 by sliding an equally narrow sized box over all bands and extracting the pixel intensities. For Protein co-IPs see Text S1. GeneBank Accession Number of GLS-1: FJ610055.

## Supporting Information

Text S1 Supplemental Materials and Methods.
(0.07 MB DOC)
