# Supplementary material for: GLS-1, a Novel P Granule Component, Modulates a Network of Conserved RNA Regulators to Influence Germ Cell Fate Decisions
Source: PLoS Genet. 2009 May 22;5(5):e1000494. doi: 10.1371/journal.pgen.1000494 (PMC2679207; doi:10.1371/journal.pgen.1000494)
Supplement: Text S1 — Supplemental Materials and Methods. (0.07 MB DOC) [file pgen.1000494.s001.doc]

## Supplemental Materials and Methods

### Strains

The *gls-1(ef4)* allele is an in-frame deletion that also carries a point mutation in exon 7, encoding a GLS-1 protein missing aa384-692 with a P362L change. The *gls-1(ef8)* allele generates a C-terminally truncated protein coding mRNA, covering aa1-248 of GLS-1 with a novel 63aa long C-terminus. Both mutations were out-crossed ten times against wild-type. Primers used to isolate and follow the mutation are available upon request. Mutations linked to LGI were balanced over rearrangement *hT2g [qIs48 (I;III)]* and mutations on LGII were balanced over *mIn1 [mIs14 dpy-10(e128)]*. We generated homozygote *gls-1; fbf-1; unc-15 fog-2* animals by creating a *gls-1(ef8)/ccIS4251; fbf-1(ok91)/mIn1; unc-51(e1189) fog-2(q71)/+* strain and maintaining it by following the closely linked *unc* phenotype to *fog-2*. *ccIS4251* is a GFP transgene closely linked to *unc-15(e73)* on LGI.

### RNAi constructs and treatment

For RNAi experiments cDNAs specific for *gls-1*, *gld-4*, *fem-1*, *fem-2*, *fem-3*, *fog-1*, *fog-2* and *fog-3* were subcloned after RT-PCR amplification into pPD129.36 (pL4440) and sequenced. Double stranded RNA was either administered through gonad injections into young adult animals for *gls-1* and *gld-4* at a concentration of 1-2μg/μl after *in vitro* transcription or through feeding L4 larvae with *E. coli HT115 [DE3]* carrying the RNAi feeding constructs for *fem-1*, *fem-2*, *fem-3*, and *fog-1* according to [1]. The F1 generation was scored for phenotypes and is expected to knock down maternal and zygotic gene product in the case of gonad injections.

### Yeast Two-Hybrid Analysis and *gls-1* cDNA analysis

A yeast 2-hybrid screen was carried out with full-length (FL) GLD-3L and GLD-3(aa 57-969), deleted for the GLD-2 interaction domain, as a bait in the L40 yeast strain using 5mM 3-amino-1,2,4-triazole. Five partial *gls-1* cDNAs were recovered in each screen. A reverse yeast 2-hybrid screen was carried out with GLS-1(FL) as a bait and 54 clones of partial *gld-3* cDNAs were recovered.

We used 5’RACE from adult worms to clone full-length C36B1.8 mRNAs and found all cDNAs *trans*-spliced exclusively to SL2. The longest *gls-1* transcript is 3712nt in length. A second alternatively spliced transcript is marginally shorter and misses 6nt at the 3’ end of the end of the first exon. The physical distance between *dhfr-1* and *gls‑1* in *C. elegans* is less than 200 nucleotides, which is in good agreement with the majority of genes in operons [2]. An alternative splice form of *gls-1*, affecting the second exon eliminating two amino acids was also detected. Our genomic analysis of the *gls-1* locus in *C. remanei* and *C. briggsae* was aided by their genomic sequences available at www.wormbase.org and the NCBI sequence databases. We confirmed the expression, SL2 trans-splicing and the overall cis-splicing pattern of *gls-1* in both species by RT-PCR of total RNA preparations of adult worms.

For binding domain identification full-length or partial GLS-1 or GLD-3 proteins were fused to the LexA DNA-binding domain using a derivative of the vector pBTM116. GAL4 activation domain fusions were created using derivatives of the pACT vector and co-transformed with pBTM116 clones in the yeast host strain L40. Beta-galactosidase quantifications and filter lifts were processed as described [3]. GLD-3ax562 interactions were tested for temperature sensitivity by growing the co-transformed yeast cells at 16°C and 30°C; GLD-3ax562 failed to bind specifically to GLS-1 at both temperatures. All protein fusions were verified for expression by immunoblotting using antibodies from Santa Cruz (anti-GAL4 and anti-LexA).

### Immunoprecipitations

For each co-immunoprecipitation from SF+ cells 500μl of frozen extract was pre-cleared at 16000g for 15 minutes at 4°C. All subsequent biochemical steps were carried out on ice. Proteins were isolated from the supernatant using antibodies coupled to Protein A-agarose beads for 1 hour. Beads were washed 5x in 2xPBS containing 0.2% X-100 and boiled in 50μl of 2xSDS sample buffer. 1/5 of the IP was separated on a 8% SDS/PAGE gel. Equal protein expression was determined by immunoblotting and subsequent probing with penta-His (Qiagen) and Maltose-Binding-protein (NEB) antibodies.

Immunoprecipitation experiments from worm extracts were carried out essentially as described in [4] using a modified worm lysis buffer [50mM Hepes-KOH pH7.4, 70mM KAc, 1mM NaF, 20mM β-glycerolphosphate, 5mM MgOA, 0.1% Triton, 10% Glycerol]. Precipitates were eluted with SDS-PAGE sample buffer and separated by SDS gel electrophoresis followed by Western blotting. Monoclonal antibodies were purchased from Santa Cruz (anti-GFP) and Sigma (anti-tubulin and anti-actin).

For RNAse A treatment, 0.1mg RNase A (Sigma) was added to 1 ml extract and pre-incubated at 4 degrees for 15 minutes on a wheel. This extract was subsequently incubated with antibody-coated beads as described for the non-RNase A treated samples. A denaturing Agarose gel was run to verify the effectiveness of the RNase A treatment compared to a mock treated sample.

## Supplemental References:

1. Kamath RS, Martinez-Campos M, Zipperlen P, Fraser AG, Ahringer J (2001) Effectiveness of specific RNA-mediated interference through ingested double-stranded RNA in Caenorhabditis elegans. Genome Biol 2: RESEARCH0002.

2. Blumenthal T, Evans D, Link CD, Guffanti A, Lawson D, et al. (2002) A global analysis of Caenorhabditis elegans operons. Nature 417: 851-854.

3. Eckmann CR, Kraemer B, Wickens M, Kimble J (2002) GLD-3, a bicaudal-C homolog that inhibits FBF to control germline sex determination in C. elegans. Dev Cell 3: 697-710.

4. Eckmann CR, Crittenden SL, Suh N, Kimble J (2004) GLD-3 and control of the mitosis/meiosis decision in the germline of Caenorhabditis elegans. Genetics 168: 147-160.
